# Hardware Acceleration of Digital Pulse Shape Analysis Using FPGAs

**DOI:** 10.3390/s24092724

**Published:** 2024-04-25

**Authors:** César González, Mariano Ruiz, Antonio Carpeño, Alejandro Piñas, Daniel Cano-Ott, Julio Plaza, Trino Martinez, David Villamarin

**Affiliations:** 1 Instrumentation and Applied Acoustic Research Group, Universidad Politécnica de Madrid, 28031 Madrid, Spain; mariano.ruiz@upm.es (M.R.); antonio.cruiz@upm.es (A.C.); a.pinas@upm.es (A.P.); 2 Centro de Investigaciones Energéticas, Medioambientales y Tecnológicas (CIEMAT), 28040 Madrid, Spain; daniel.cano@ciemat.es (D.C.-O.); julio.plaza@ciemat.es (J.P.); trino.martinez@ciemat.es (T.M.); david.villamarin@ciemat.es (D.V.)

**Keywords:** digital pulse shape Analysis, FPGA, JESD204B, Xilinx HLS, MicroTCA, hardware acceleration

## Abstract

The BC501A sensor is a liquid scintillator frequently used in nuclear physics for detecting fast neutrons. This paper describes a hardware implementation of digital pulse shape analysis (DPSA) for real-time analysis. DPSA is an algorithm that extracts the physically relevant parameters from the detected BC501A signals. The hardware solution is implemented in a MicroTCA system that provides the physical, mechanical, electrical, and cooling support for an AMC board (NAMC-ZYNQ-FMC) with a Xilinx ZYNQ Ultrascale-MP SoC. The Xilinx FPGA programmable logic implements a JESD204B interface to high-speed ADCs. The physical and datalink JESD204B layers are implemented using hardware description language (HDL), while the Xilinx high-level synthesis language (HLS) is used for the transport and application layers. The DPSA algorithm is a JESD204B application layer that includes a FIR filter and a constant fraction discriminator (CFD) function, a baseline calculation function, a peak detection function, and an energy calculation function. This architecture achieves an analysis mean time of less than 100 µs per signal with an FPGA resource utilization of about 50% of its most used resources. This paper presents a high-performance DPSA embedded system that interfaces with a 1 GS/s ADC and performs accurate calculations with relatively low latency.

## 1. Introduction

BC501A liquid scintillators are commonly used in nuclear physics applications. These detectors can simultaneously detect gamma radiation and neutrons. Upon the incidence of such a particle, ionization of the liquid occurs, and the de-excitation produces light in the ultraviolet and blue part of the visible spectrum. The light is then collected by a photomultiplier and converted into an electric pulse. The pulse shape produced by gamma photons differs from those produced by neutrons, and thus, an analysis of the pulse allows discrimination between both kinds of particles [1,2]. Guerrero et al. developed an improved method of digital pulse shape analysis (DPSA) [3], which is based on the integration of two different ranges of the signal. The relationship between the charge collected in these two ranges allows for the identification of the pulse as produced by a gamma photon or by a neutron. The use of digital electronics and DPSA offers a more versatile solution than hardware-based analog-to-digital or charge-to-digital converters. There are different approaches to implementing the DPSA to discriminate neutrons and gamma, as summarized in [4]. The majority of the solutions are based on digitizing the signals and implementing offline analysis applications using a computer. Additionally, there are emerging approaches that employ machine learning techniques for offline analysis and even real-time analysis using neural networks in data acquisition (DAQ) devices. In both cases, the amount of digitized data to be transferred to the computer can become a bottleneck when the number of channels and count rates in the detectors are large. As an alternative, the required calculations for the DPSA can be performed with reduced latencies by utilizing field-programmable gate arrays (FPGAs) and hardware process acceleration techniques, which are extensively used (see [5]). This potentially enables real-time applications such as time-of-flight (ToF) measurements related to the measurement of neutron spectra in various types of nuclear physics experiments, especially under high count rate conditions [6,7]. However, the possibility of real-time analysis is highly dependent on the available FPGA resources and the algorithm’s complexity.

This paper proposes an FPGA-based hardware implementation for DPSA, using floating point operations, of signals from a BC501A liquid scintillator. The signals are digitalized at a rate of 1GS/s with 16-bit resolution for use in a real-time analysis system.

**Figure 1:**
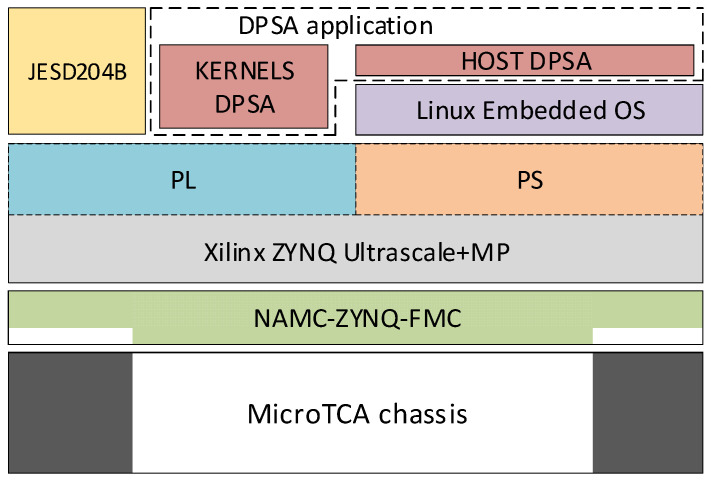
Hardware elements, OS, and DPSA application stack.

The system is based on a Micro Telecommunications Computing Architecture (MicroTCA) chassis [8,9,10], which provides physical, mechanical, electrical, and thermal support for a NAMC-ZYNQ-FMC Advanced Mezzanine Card (AMC) [11]. The NAMC-ZYNQ-FMC is a system-on-a-chip (SoC)-based AMC whose main component is a Xilinx ZYNQ Ultrascale + MP SoC [12]. The JESD204B standard [13,14] is implemented in the SoC programmable logic (PL) area to interface the digitizer and the DPSA application. This standard defines a serial interface that connects high-speed converters to logic devices such as FPGAs and Application Specific Integrated Circuits (ASIC). The JESD204B standard is implemented to connect the ADC directly to the processing hardware. Analog Devices provides an open-source intellectual property (IP) framework distributed under the GPL2 license to implement the JESD204 interface and the software to configure all the hardware elements [15]. Figure 1 displays the stack of the hardware and software elements mentioned earlier. The bottom two layers of the stack show the MicroTCA chassis that supports the NAMC-ZYNQ-FMC board. The third layer from the bottom features the Xilinx ZYNQ Ultrascale + MP SoC, which integrates a high-performance ARM-based multicore multiprocessing system (PS) with ASIC-class PL.

The objective of this study is to evaluate the suitability and limitations of the MicroTCA platform for its use in a data acquisition system, with real-time analysis of the detector signals using a realistic pulse shape analysis algorithm.

Guerrero et al. [3] state that the DPSA aims to extract the physically relevant parameters for the BC501A detector. The parameters include the time at which the pulse occurs, amplitude, and integrals over specific time intervals to determine the type of incident particle. The DPSA application layer, located at the top of the stack in Figure 1, achieves this goal. The Xilinx ZYNQ Ultrascale + MP SoC within the processing system (PS) uses a Linux-embedded system to execute the DPSA application. This application coordinates kernel execution and reads the analysis results. The hardware kernels responsible for signal analysis and achieving the DPSA’s objectives are located in the SoC PL area. On the top layer of Figure 1, the JESD204B interface is shown on the left. The DPSA kernels are also part of the application layer for the JESD204B standard.

Key contributions of the work are the use of floating-point-based hardware to achieve maximum accuracy in the estimation of the relevant parameters, the complete implementation of the DSPA algorithm using high-level synthesis (HLS) in a MicroTCA platform, and the measurement of the performance. A positive result can impact the design of the next generation of data acquisition systems to be used in nuclear physics experiments since it would combine the versatility of algorithms programmed in a high-level language with real-time analysis in high-count rate applications. To verify the implementation, nearly 400,000 signals digitalized from a BC501A and stored in a database were analyzed [16].

## 2. Materials and Methods

### 2.1. MicroTCA

MicroTCA is an open-source standard developed by the PCI Industrial Computer Manufacturers Group (PICMG) [17]. It provides a modular and scalable computing architecture to build robust and high-performance systems in a small form factor. A typical MicroTCA system consists of up to twelve AMCs, a MicroTCA Carrier Hub (MCH), power modules, and cooling units, all connected through the backplane. The MicroTCA chassis is the enclosure that provides physical support to the system components, while the MCH is responsible for overall management [18].

### 2.2. NAMC-ZYNQ-FMC

The NAMC-ZYNQP-FMC is an FPGA-based FMC carrier AMC. Its key component is a Xilinx Zynq UltraScale+ MPSoC that provides programmable acceleration and heterogeneous processing through the following elements:

- Quad-core ARM Cortex-A53 for application processing.
- Dual-core ARM Cortex-R5 for real-time processing.
- ARM Mali-400MP2 GPU.
- FPGA Programmable Logic.

**Figure 2:**
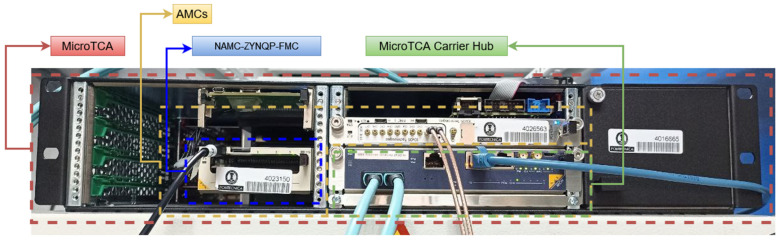
MicroTCA with three AMC boards including the NAMC-ZYNQ-FMC.

Figure 2 shows the MicroTCA chassis with three AMC boards, including the NAMC-ZYNQ-FMC.

### 2.3. JESD204B Implementation

The JESD204B implementation on the Xilinx Ultrascale+ MPSoC consists of the FPGA design implemented in the PL using hardware description language (HDL) for the physical and data link layers and HLS for the transport and application layers. An embedded Linux distribution running on the PS is used to configure the peripherals implemented in the PL and to support the host software. These are based on the four layers of functionality defined by the JESD204B specification, as described by Harris and Fan in [19,20], respectively. The letter B in the JESD204B standard refers to its second revision. This revision defines links with multiple synchronized lanes with lane rates up to 12.5 Gbps, which are requirements to achieve deterministic latency. It ensures that link latency is repeatable between power cycles and link resynchronization.

**Figure 3:**
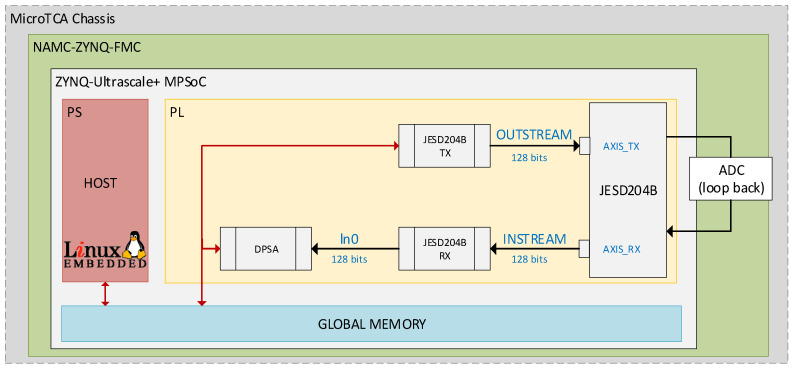
DPSA implementation scheme. The PL implements the DPSA using the streaming data read with the JESD204B interface. An external loopback connects the signal generation with the data acquisition.

The JESD20B_TX kernel, which is a component of the JESDB204B transport layer, is utilized to stream data and emulate a DAC. The transmitter’s output is connected to a JESD204B receiver that emulates the ADC. This method eliminates the need to implement and validate the processing algorithm in the FPGA using a physical ADC. An external loopback has been used to connect the JESD204B DAC and JESD204B ADC hardware elements. Initially, the signals are stored in a file on the host system. The host transmits signals to the global memory accessible by the FPGA PL. The JESD204B_TX kernel reads the data signals from the global memory and streams them to the subsequent kernels. Figure 3 shows the DAC and ADC transmitter and receiver interfaces of the JESD204B connected by an external loopback, the implemented kernels on the PL, and the interactions of the PS and PL with the global memory. The transport and application layers are implemented in HLS. The JESD204B_TX, JESD204_RX (transport layer), and DPSA (application layer) kernels are located outside of the JESD204B block.

**Figure 4:**
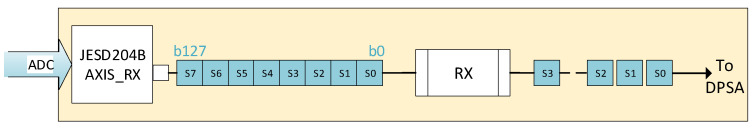
Adaptation of the 1GS/s ADC to 125 MHz kernel clock reading 8 samples (S0–S7).

Since the ADC acquisition sampling rate and resolution are preset parameters, the JESD204B is designed to be coupled according to the FPGA resources. The JESD204B clock frequency is set to 125 MHz with a 128-bit data frame. In this scenario, 8 samples are acquired per system clock cycle, with each sample having a resolution of 14 bits (2 bytes wide). Therefore, 16 bytes are sent in a clock cycle. Figure 4 shows the data stream that couples the difference between the data acquisition and processing clocks.

### 2.4. DPSA Application

**Figure 5:**
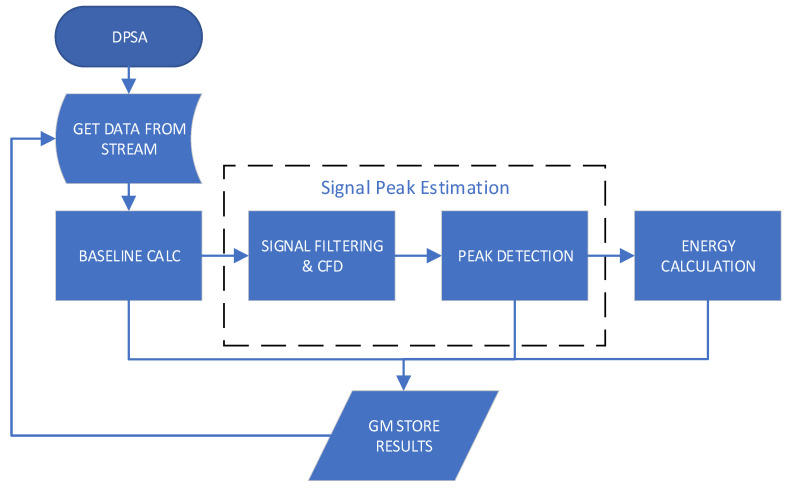
DPSA application flowchart.

The DPSA kernel is implemented on the application layer of the JESD204B to achieve its objectives. Figure 5 shows the flowchart of the DPSA kernel, which operates using the pipeline technique. The kernel’s functions obtain the data buffer for the stream, calculate the baseline, filter the signal, detect the peak, and calculate the energy (see Supplementary Materials Section).

#### 2.4.1. Load Input Data from Stream

At the start of the kernel execution, a function loads these data and identifies the signal’s area of interest to reduce computing time in subsequent kernel functions. The region of interest is identified using a threshold and starts n samples before the trigger point (start point). The threshold value and n are determined by the hardware configuration. If no additional pulse is detected, the end point of the signal of interest is the starting point plus a time interval, also determined by the hardware configuration. If another pulse is detected, the region of interest includes the entire signal from the starting point. Each loaded sample is 2 bytes wide.

#### 2.4.2. Baseline Calculation

**Figure 6:**
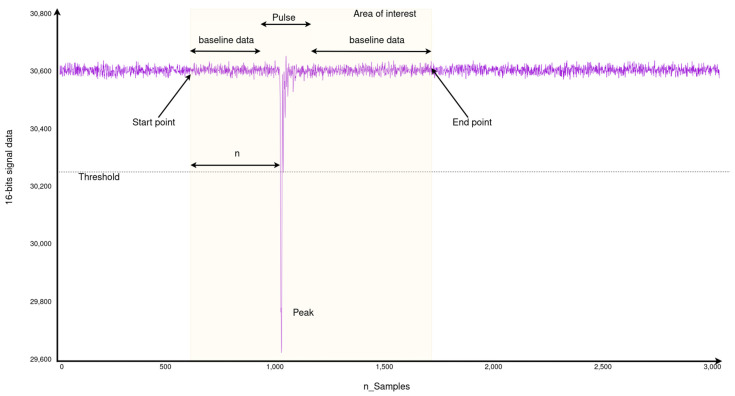
Example of an Input signal highlighting the interest area for the analysis with the main relevant parameters.

The ADC provides a 14-bit resolution number for the signal baseline. To enhance the accuracy, a new baseline value is calculated by analyzing the signal around the detected peak. This involves considering the signal span before and after the peak, from the starting point to the peak start, and from the end of the peak to the end signal, respectively (Figure 6). Since the peak width is a preset parameter, detecting the spans to calculate is straightforward. The average of the signal spans with the least variance is used to determine the baseline. In this function, the standard deviation is also calculated.

#### 2.4.3. Signal Peak Estimation

Two functions were developed to estimate the number of peaks in each signal, determine the time at which each pulse occurs, and identify whether the signal has pile-ups. The time at which each pulse occurs is calculated using the constant fraction discriminator (CFD) method [21,22] after applying a finite impulse response (FIR) filter. This time represents the duration between the onset of the signal and the occurrence of the pulse. The first function, labeled Signal Filtering and CFD in Figure 5, applies low-pass filtering to the signals using a 20-tap FIR filter defined in Equation (1), where *X_n_* and *Y_n_* are the input and output samples, respectively. The decision to use a 20-tap FIR filter is based on the fact that the values decrease with each iteration, and values beyond 20 have a negligible impact on the final result. The host computes the FIR filter coefficients, which are then loaded by the kernel at the start. The coefficients (*h_i_*) are calculated using Equation (2), where the *rc* value is preset.

(1) $$Y_{n}=\sum_{i=0}^{20}h_{i}X_{n-i}$$

(2) $$h_{i}=\left(1-e^{-1/rc}\right)\times e^{-i/rc}$$

The CFD is applied in conjunction with the FIR filter. In this phase, the filtered signal *Y* is multiplied by a factor (*f* in Equation (3)) and then subtracted from the same delayed signal (defined as *Y_n-delay_*). Equation (3) describes the process of obtaining the CFD signal. Both the *f* and the *delay* are predetermined values.

The resulting output of this function comprises a filtered signal and a CFD signal, which are then forwarded to the subsequent function.

(3) $$Y_{n-delay}=Y_{n-delay}-f\times Y_{n}$$

The second function, labeled as Peak Detection in Figure 5, identifies the precise point at which a peak occurs. This is determined by the point at which the CFD signal crosses the baseline. A threshold is also applied to the filtered signal to discard noise signals that may be incorrectly detected as a valid signal.

To ensure accuracy, interpolation is necessary to obtain the exact point at which the CFD signal crosses the baseline. The resulting response is a floating-point number that indicates at which point the pulse occurs and is less than the previously detected sample number. Equation (4) determines the point at which the signal CFD crosses the baseline (*Z_PULSE_*), where *i* is the sample number at which the filtered signal crosses the baseline.

(4) $$Z_{PULSE}=i-1+\frac{{CFD}_{i-1}}{{CFD}_{i}-{CFD}_{i-1}}$$

To be recorded as one of the analysis results, these data must first be converted to time values by multiplying it by the sample rate (1 GS/s) and subtracting any pre-trigger delay.

(5) $$T_{PULSE}=Z_{PULSE}\times SampleRate-PreTriggerDelay$$

**Figure 7:**
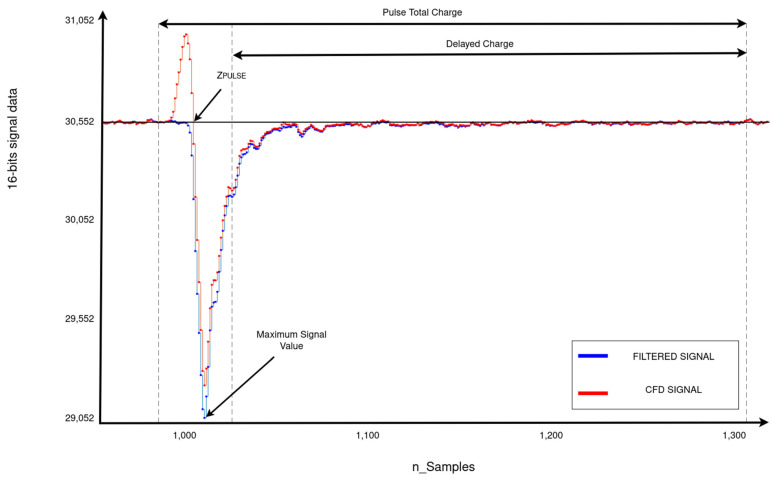
Close-up of the peak of the function output signals. The blue signal represents the filtered signal, while the red signal represents the *CFD* signal. The point where the pulse occurs is marked as *Z_PULSE_*, the total range to be integrated is marked as Pulse Total Charge, and the remaining range of the pulse is marked as Delayed Charge.

Figure 7 displays a close-up of the peak of the filtered signal and the *CFD* signal, with *T_PULSE_* indicating the time of the pulse.

The function also detects the number of peaks per signal and identifies pile-ups. It is possible to detect multiple peaks in some signals. A flag is used to increase the number of the detected peaks when the filtered signal goes above the threshold, indicating that this part of the signal is not part of a peak. The flag prevents this increase as long as the signal remains below the threshold, indicating that this part of the signal is part of a peak.

Finally, if multiple peaks are detected and they overlap, a pile-up occurs. Handling undershoots, rebounds, and high-order pile-ups requires a more complex algorithm. However, detecting signal interference from pile-ups is possible and within the scope of this project.

#### 2.4.4. Energy Calculation

This function calculates the energy array for each valid pulse. The first element of the array contains the maximum signal value. The remaining elements represent the signal charge, which is obtained by integrating the signal over two time intervals. The first interval is a preconfigured range around Z_PULSE_, and the second interval is from the remaining range of the pulse, which represents the “delayed charge” [2]. Finally, these two integrals are added to obtain the integral of the total pulse duration. Figure 7 shows the times for the total and delayed charges.

#### 2.4.5. Global Memory Store Results

The PS area of the Xilinx Ultrascale+ MPSoC PS area runs the host software, which is written in C++ and cross-compiled for ARM architecture. The software runs on an embedded Linux deployed with Petalinux. The host software has three main functions: reading signals from a file and writing them to a buffer that the PL can access, coordinating the execution of kernels on the PL, and reading the results stored by the kernels in global memory (GM). The Xilinx Zynq Ultrascale+ MPSoC contains various memory components that are accessible throughout the system, as detailed in [23], known as GM. Two 8GB onboard DDR4 (Double Data Rate) memory chips are used to store data for processing.

The final function of the PL side DPSA kernel is to write the analysis results in GM, allowing the host to access them. An array of analysis results is stored for each pulse.

### 2.5. Results Analysis

To evaluate the reliability and performance of the system, nearly 400,000 signals that were digitalized from a BC501A detector were analyzed. The DPSA generates an array of results for each signal, which is saved in a CSV file by the host. The results were comparable to those obtained from a C++ analysis, which served as the basis for this project. Further details on performance and reliability are discussed in the Discussion section.

The performance of the kernels was analyzed using Vitis Analyzer and VIVADO tools to measure execution times and FPGA resource utilization. Profiling flags were enabled during kernel compilation to obtain performance information.

## 3. Results

**Figure 8:**
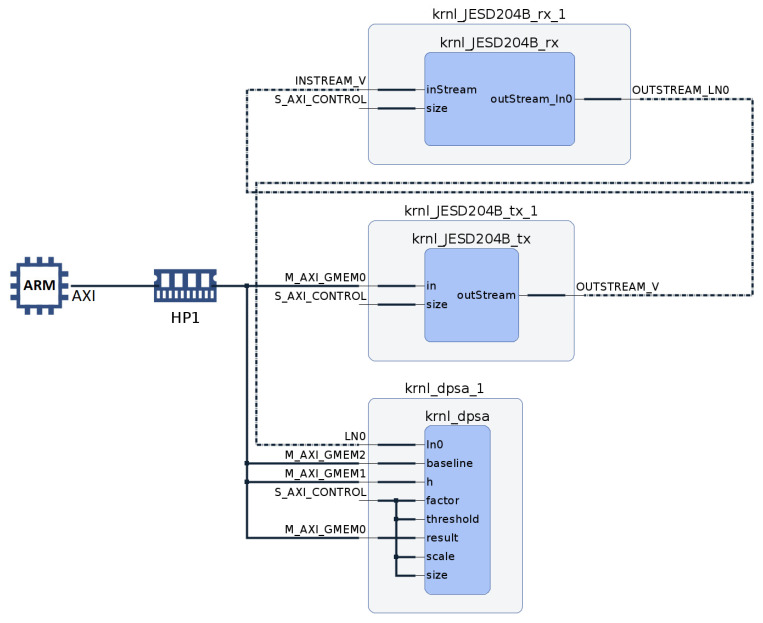
Vitis Analyzer system diagram.

This work presents a DPSA system that can achieve high performance by directly interfacing with a 1 GS/s ADC through its JESD204B interface. Figure 8, which is extracted from the Vitis Analyzer system diagram, provides a visual representation of the interconnection of kernels utilizing the Advanced eXtensible Interface (AXI). The kernels are connected through AXI streams, as shown by the dotted lines. The host connects to the Global Memory HP1 through AXI version 4 (AXI4), as indicated by the solid lines. Communication from the host to kernels is established using the simplified AXI4-LITE protocol.

### 3.1. FPGA Used Resources

Table 1 displays the FPGA resource utilization for the JESD204B implementation and the DPSA application. The DPSA application consists of the DPSA kernel and the JESD204B TX and RX kernels. The lookup tables (LUT) on JESD204B and DPSA application are the most heavily utilized resources, reaching 19.26% and 25.75%, respectively. The JESD204B resources refer to the utilization of the board support package (BSP), which includes the JESD204B and all necessary software components and drivers to support the platform.

### 3.2. Performance

To evaluate the system’s performance, nearly 400,000 signals digitalized from a BC501A detector were processed. Each signal consists of 3000 samples of 14-bit data. Table 2 displays the execution times for each kernel, as well as the frequency at which the process was performed. This frequency differs from the data transfer, which previously operated at 125 MHz, as it is the processing clock.

The execution times of the DPSA, which is responsible for the computation process, exhibit low variance. However, the kernels of the JESD204B transport layer exhibit significant differences between minimum, average, and maximum times. The purpose of the JESD204B transport layer kernels is to transmit data via stream to the DPSA kernel for processing and not to perform any computations. The execution times of the JESD204B transport layer kernels vary due to their dependence on the CPU and the DPSA kernel data readout (see Section 4.2). It is important to note that these data are read from a previously obtained file and are performed by the CPU. Despite this, the low variance in the DPSA kernel is satisfactory. What is relevant is the time used by the DPSA kernel (see Section 4.3).

The parameters to process the signals and the signals are sent by the host to the PL area through global memory. These parameters are referred to as preset parameters throughout the text. The process results are then read from the host’s global memory. Table 3 displays the transfer characteristics between the host and the global memory.

Kernels also communicate with the host by reading and writing data from global memory. Table 4 shows the characteristics of these transfers. Each kernel reads at least once, but only the DPSA writes results to global memory.

### 3.3. Accuracy

The accuracy of the DSPA algorithm implemented in the FPGA is tested by processing the 398,960 signals and comparing the results with the C++ application. Table 5 shows the results obtained with one single signal for the relevant algorithm’s parameters. Section 4.2 discusses more details.

## 4. Discussion

In the discussion section, three points should be considered: how the algorithm was optimized to reduce computational costs and achieve low latencies, what causes the accuracy errors presented in Section 3.3, and whether the system can be considered a real-time system or not.

### 4.1. Algorithm Optimization

The algorithm has been optimized to create a high-speed processing system. It is unnecessary to process the entire signal since there is a significant amount of noise present around the pulses, which are the focal points of interest. This significantly increases computational cost, with 20 operations performed per sample in the FIR filter step. To address this issue, the function checks if the signal has passed the threshold that indicates a pulse has occurred while the input signal buffer is being filled from the input stream. Subsequent functions utilize this value to restrict the signal. The pulse width is a predetermined value that corresponds to the typical pulse width of this experiment. Therefore, the signal is also limited at the end of the pulse.

### 4.2. Accuracy Error

The issue arises from differences in implementation approaches. In the C++ implementation, the baseline is calculated twice. The entire signal is initially used except for the points whose variance exceeds a given value. This baseline is then used for subsequent calculations. A new value for the baseline is then calculated using only the contiguous sections of the pulse. This new value and the variance are presented as the result.

To reduce computation time and the FPGA resources used in the hardware implementation, the baseline is calculated only once using the contiguous sections of the pulse. Therefore, these baseline data and standard deviation in the table are precise. The calculated data is then used in the following functions.

Table 6 displays the mean and the standard deviation of error of the relevant parameters after processing the 398,957 signals, which exhibited negligible differences.

### 4.3. Real-Time System

Determining whether a system is real-time depends on the criteria used. It is important to note that the system’s latency is equivalent to that of the DPSA kernel, where the calculations are performed. Execution times for JESD204B TX and RX kernels are related to the global memory reading times of the signals. These signals are used solely to simulate signal generation and data acquisition behavior in conditions similar to the real experiment, facilitating prompt algorithm validation. Table 2 in Section 3.2 displays the execution times of the DPSA kernels. The maximum value is 134 µs, and the average is 99 µs, indicating stable values.

### 4.4. Potential Application

This work was aimed at potential applications in the nuclear science field, where data acquisition systems (DAQs) have traditionally relied on analog electronics. However, the possibility of directly digitizing the signals coming out of the detectors allows us to reduce the experimental setups, to better evaluate issues such as dead time and pile-ups, and to integrate a large number of channels.

FPGAs are increasingly being integrated into digital commercial of the self (COTS) DAQ products [24,25,26], allowing online analysis of signals. In scenarios using multiple channels and a high trigger rate, there is a need for fast processing while yielding accurate values of the integrated charge, as well as accurate values of the timing of the signals, which is important in the case of time-of-flight experiments to measure neutron energy spectra. Some of these COTS allow the integration of HLS blocks to implement customized applications [26].

### 4.5. Comparison with Other State-of-the-Art Works

To highlight the implementation achieved it is important to compare with other similar solutions published recently. The solution presented in [27] works at 250 MHz and achieves a better event rate but uses a fixed-point calculation method. The hardware solution used is a standalone, noncommercial product. The work described in [28] also uses a MicroTCA platform; the hardware design in the FPGA is implemented in Verilog, reaching 2 Mevents/s but using fixed-point calculations.

## 5. Conclusions

This paper presents the implementation of a pulse analysis application for a scintillator using hardware acceleration techniques (based on HLS) using a high-speed data acquisition and processing system based on MicroTCA technology. The system comprises an AMC with an FPGA that implements a JESD204B interface with the ADCs. The obtained results confirm that the implementation can be integrated into data acquisition systems to obtain real-time results under specific experimental conditions. Table 1 in Section 3.1 details the utilization of FPGA resources for the entire implementation. The Lookup Tables (LUTs) are the most utilized element, accounting for 45.01%. In terms of performance, the system can process a signal digitalized at 1 GS/s with 3000 samples in a mean time of 100 µs, resulting in a sustained processing rate without losses of 10^4^ counts per second. Additionally, it is worth highlighting that using the JESD204B interface to emulate the signal generation in conditions such as the real experiment enables prompt algorithm validation. The execution time and latency results demonstrate the possibility of integrating the solution on real experimental platforms using tools that enable firmware adaptation of the acquisition devices. For comparison, the same rate per detector in the data acquisition system described in [29], with a configuration of 32 channels (6 cards with 4 channels), would require a sustained transfer rate between the cards and PCIe digitizers of 1.36 Gbytes/s. Such a figure is not achievable with PCIe 3.0 and would require a much faster data bus. Finally, as the completed DPSA application is developed in HLS, the development time has been reduced notability.

## Figures and Tables

**Table 1:** Compute units’ resource utilization.

	JESD204B *	DPSA Application	Total
LUT (% Used)	44,374 (19.26%)	59,332 (25.75%)	103,706 (45.01%)
Register	-	82 054	82 054
BRAM (% Used)	74.50 (23.88%)	28 (8.97%)	102.5 (32.85%)
DSP (% Used)	3 (0.17%)	39 (2.26%)	42 (2.43%)

* This includes the Board Support Package (BSP), which comprises the JESD204B and all the software components and drivers necessary to support the platform.

**Table 2:** Execution times reported by the VITIS profiler tool after the computation of 398,960 signals.

	DPSA	JESD204B TX	JESD204B RX
Calls *	398,960	398,960	398,960
Total Time (ms)	39,621.6	45,460.8	27,244.7
Min Time (µs)	98	12	15
Avg Time (µs)	99	114	68
Max Time (µs)	134	13,213	6635

* The number of calls to kernels is equal to the number of signals processed.

**Table 3:** Data transfer, Host to global memory.

Transfer Type	Number of Buffer Transfers	Transfer Rate (MB/s)	Avg Size (KB)	Total Time (ms)	Avg Time (µs)
READ	39,896	17.18	0.40	929.02	23
WRITE	797,854	113.29	3.04	21,438.90	27

**Table 4:** Data transfer, Kernels to global memory.

Kernel	Transfer Type	Number of Buffer Transfers	Avg Bytes per Transfer	Total Transfer Rate (MB/s)
JESD204B TX	READ	24	250	1200
DPSA	WRITE	1	4	4800

**Table 5:** Comparison of the results obtained with the C++ application and the hardware implementation for one signal.

Result	C++	Hardware
Baseline (mV)	−10.3916	−10.3916
STD baseline (mV)	0.663682	0.663682
Peak detection time (ns)	3.600263	4.2582
Peak max (mV)	56.2582	56.2582
Total Charge (ADC counts)	556.16	563.12
Delayed Charge (ADC counts)	99.38	96.12

**Table 6:** Comparison between the results produced by the C++ application and the hardware implementations of 398,957 signals. Mean and standard deviation of the difference between both methods for relevant parameters.

Result	Diff Avg.	Diff Stdv.
Baseline (mV)	0.0006	0.024
STD baseline (mV)	0.0060	0.019
Peak detection time (ns)	0.8345	0.787
Peak max (mV)	0.0048	1.622
Total Charge (ADC counts)	5.6024	25.523
Delayed Charge (ADC counts)	8.997	37.498

## Data Availability

The research dataset can be found at https://github.com/i2a2/DPSA.

## Supplementary Materials

The following supporting information can be downloaded at: https://github.com/i2a2/DPSA, Application source code: DPSA; C++ implementation results: output_c++.csv; Hardware implementation results: output_hls.csv; https://github.com/i2a2/namc_zynqup_fmc_bsp/tree/ad_jesd204_2021.1, BSP for NAT-AMC-ZYNQUP-FMC: namc_zynqup_fmc_bsp.
